# The neurological manifestations of COVID-19 – A case series

**Published:** 2020-10-17

**Authors:** Pranav V. Karambelkar, Chaitanya S. Rojulpote, Faryal Saeed, Christina Pichiarello, Nikul Patel, Amit Sharma

**Affiliations:** ^1^The Wright Center for Graduate Medical Education, Scranton, PA, USA; ^2^Geisinger Community Medical Center, Scranton, PA, USA

**Keywords:** COVID-19, seizures, SARS-CoV, neurological invasion

## Abstract

Severe acute respiratory syndrome (SARS) is a fatal respiratory illness caused by the coronavirus (CoV). The first known case was reported in 2002, later coined as SARS-CoV. Over the last two decades, the CoV has periodically emerged in the general population, causing a varying degree of pneumonia. The most recent outbreak, now known as coronavirus disease of 2019 (COVID-19), has been on an exponential rise. Similar to its predecessors, COVID-19 causes a fatal form of pneumonia; however, in a small percentage of patients, COVID-19 has shown to cause neurological symptoms. Given that SARS-CoV and the new CoV strain share similar viral structures, COVID-19 may have the capability to invade the neurological system. We present a series of patients with COVID-19, the first of which presented with a seizure, whereas our second patient developed seizures during their hospital course. Neither patient had a previous history of epilepsy.

**Relevance for patients:**

COVID-19 has rapidly evolved since it was first reported and has proven to be a fatal infective process. The last several months have been challenging for the medical community as we try to understand the complexities of this virus. Clinicians have attempted to assess the most common presenting symptoms based on reported cases. The purpose of this study was to help understand how COVID-19 presents itself when the neurological system is involved. This case series describes the common and uncommon neurological manifestations of COVID-19. By doing so, we hope to provide clinicians with additional information to help diagnose COVID-19 in this unprecedented time and to also be wary of the uncommon presenting features.

## 1. Case 1

This is a 32-year-old gentleman with sleep apnea presented with cough, fever, shortness of breath, and reduced taste sensation. The patient worked at a nursing home and had exposure to sick contacts. On presentation, the patient had a temperature of 101 ^o^F. His chest x-ray revealed patchy bilateral infiltrates. Computed tomography of the chest revealed bilateral ground-glass opacities. He tested positive for coronavirus disease of 2019 (COVID-19) and was subsequently started on hydroxychloroquine. On day 2 of hospitalization, the patient developed acute respiratory failure requiring intensive care. The patient had two episodes of generalized tonic-clonic seizure activity, each lasting approximately 2 min, after which he was subsequently intubated. The patient required intravenous anti-epileptic medications during his course on the mechanical ventilator. He was treated symptomatically and was gradually weaned off the ventilator after 3 days. The patient was ultimately discharged from the intensive care unit after being deemed hemodynamically stable.

## 2. Case 2

This is an 82-year-old gentleman with a medical history of chronic systolic heart failure, atrial fibrillation, and hypertension who presented with shortness of breath, generalized weakness, and tremors. The patient had recently returned from Ireland. Shortly after his trip, he experienced difficulty breathing, which was associated with generalized weakness and tremors in both hands. His laboratory values were significant for a pro-BNP of 1,640, platelets of 97,000, and acute kidney injury. Computed tomography of the chest revealed bilateral ground-glass opacities. The patient was admitted to the intensive care unit in view of severe respiratory distress. He was found to be positive for COVID-19. On day 2 of hospitalization, he required mechanical ventilation for acute hypoxic respiratory failure. Two days after being mechanically ventilated, the patient had an episode of generalized tonic-clonic seizures. His seizure was terminated with lorazepam, additionally sedated with propofol, and was administered a loading dose of levetiracetam. He underwent computed tomography of the head in light of his supratherapeutic INR, which was negative for intracranial hemorrhage and did not reveal evidence of intracranial masses, lesions, or a stroke. The patient required further escalation of anti-epileptic therapy, as he developed a second isolated seizure episode the following day. Given the limitations of staff members and equipment, we were unable to obtain an encephaloelectrocardiogram. Unfortunately, the patient succumbed to septic shock despite optimal medical therapy.

## 3. Discussion

The coronavirus (CoV) is an enveloped RNA virus, commonly known to affect the respiratory and gastrointestinal systems [1]. There are numerous CoV strains, a majority of which cause self-limiting respiratory illnesses [1]. The emergence of fatal strains over the last two decades has driven the search for understanding the pathophysiology of the CoV. Severe acute respiratory syndrome CoV (SARS-CoV) was first reported in China in 2002 [2], causing a highly pathogenic atypical pneumonia, later found to originate from a zoonotic source. In 2012, the middle eastern respiratory syndrome CoV was documented to have a similar potential to SARS-CoV but was found to be isolated from infected camels [3]. The latest CoV species, SARS-CoV-2, has rapidly evolved into a global pandemic since it was first reported in December of 2019. Its origin was traced to be zoonotic in origin [4], similar to its predecessors.

SARS-CoV-2, later coined as COVID-19 by the WHO, predominantly presents with symptoms including fever, cough, fatigue, and dyspnea. In addition, SARS-CoV-2 produces a wide array of laboratory abnormalities, including lymphopenia, elevated c-reactive protein, sedimentation rate, D-dimer, IL-6, and various cytokine elevation [4]. A vast majority of cases rapidly progress to acute respiratory distress syndrome and necessitate the need for mechanical ventilation [4]. Although primarily a respiratory syndrome, a small percentage of patients infected by SARS-CoV-2 were found to have neurological symptoms.

A recent meta-analysis detailed various presenting signs and symptoms of COVID-19 [5]. Unspecified neurological manifestations of COVID-19 were defined as symptoms that occurred in the earlier stages of the disease. Headaches, myalgia, fatigue, nausea/vomiting, and confusion were some of the most common presenting features. Specific neurological manifestations included the loss of taste and smell, which was reported in a vast majority of patients [5]. Other neurological manifestations that have been reported include evidence of meningeal involvement, encephalitis, and Guillain-Barre syndrome [5]. An interesting case documented by Moriguchi *et al*. described a patient presenting with unexplained seizures due to COVID-19, who was diagnosed to have meningitis with positive viral isolates found in the cerebrospinal fluid [6].

Our first patient presented with common respiratory symptoms in addition to hypogeusia. The loss of taste is a subjective finding and challenging to assess clinically. We were able to rule out common etiologies related to hypogeusia, including infectious, inflammatory, vitamin deficiencies, endocrinological, and neurological etiologies [7]. The patient subsequently experienced two witnessed episodes of generalized seizure activity, and each was terminated with lorazepam. Our second patient presented with new-onset tremors involving his upper extremities bilaterally. We believe that his tremors may have been a coincidental finding associated with his generalized weakness. However, during his hospital course, he had two isolated episodes of generalized tonic-clonic seizure activity. The patient was started on scheduled lorazepam and sedated with propofol after his first seizure but required an escalation of therapy to control his subsequent seizures.

In both cases, we ruled out common etiologies of seizures, including hypoxia, metabolic abnormalities, history of substance abuse, and evidence of brain injury. Our first patient was on day 1 of hydroxychloroquine. In sporadic cases, hydroxychloroquine may potentially cause seizures, but only if taken for an extended period [8]. During the 2002 outbreak, SARS-CoV was discovered in cerebrospinal fluid [9]. Arbour *et al*. conducted an experimental study involving brain autopsy samples in patients with a history of multiple sclerosis, which revealed the presence of lesser-known CoV strains in neural tissue [10]. It is therefore rational to postulate that SARS-CoV-2 has similar potential to be present in the neural tissue of a small percentage of COVID-19 patients.

The process by which COVID-19 invades the neurological system remains unanswered. However, an understanding of the virus’s ability to spread rapidly is imperative to comprehend its capability to spread from animals to humans. After the SARS-CoV outbreak in 2002, extensive genetic research revealed angiotensin converting enzyme-2 to be the protein responsible for host attachment [11], and the spike receptor glycoprotein which played a crucial role in determining the extent of host infection [11]. SARS-CoV was confirmed to be found in neurons of autopsies of infected individuals. It was postulated that SARS-CoV may spread from the respiratory tract either through the hematogenous route or by infected immune cells [12]. Further studies have shown neurons in human brains to express ACE2 [12,13], which would further increase the viral load of SARS-CoV-2 in the human brain. A notable finding documented by An *et al*. revealed the presence of taste and smell receptors in the respiratory tract [14]. It is likely the novel CoV has the capability to selectively affect these receptors, explaining dyspnea, and hypogeusia as common presenting symptoms; however, this correlation would require further investigation.

In the earlier months of the pandemic, patients who were being admitted with COVID-19 were documented to have an overwhelming inflammatory response due to a plethora of cytokines [15]. It is possible this inflammatory response disturbs the blood-brain-barrier just enough to potentially allow the novel CoV to invade the neurological system. We can also postulate that since SARS-CoV-2 and SARS-CoV share a similar structure and function [1], it can not only invade neurons but, more importantly, lower the seizure threshold in patients. On the other hand, it is also important to consider that certain patients who presented with neurological manifestations of COVID-19 were found to have aseptic cerebrospinal fluid, indicating that further studies are required to describe its true potential to invade the neurological system. An additional method to aid diagnosis of neurological involvement included imaging. Magnetic resonance imaging in a small group of patients with unknown encephalopathic features revealed leptomeningeal and frontotemporal involvement [16]. A strong conclusion could not be reached about which imaging findings correlated with a diagnosis of COVID-19 and, hence, requires an extensive follow-up study.

Our theory was limited by the inability to confirm the actual presence of the virus in cerebrospinal fluid analysis and to proceed with electroencephalography. In light of current events and limited equipment, we based our findings on detailed patient history, thorough clinical assessment, and the clinical patterns based on SARS-CoV. It was essential to approach each patient holistically and maintain a broad list of differential diagnoses. SARS-CoV-2 is well documented to cause devastating respiratory failure, but it may also have the ability to set off a cascade of neurological complications. These theories require further studies as more cases are evaluated in this unprecedented time. We believe that additional research is required to understand the pathophysiology of this CoV variant.

### Conflicts of Interest

The authors declare no conflict of interest

### Funding

None.
